# The interplay between osteosarcopenia and intrinsic capacity: insights and associations with all-cause mortality in the Toledo Study for Healthy Aging

**DOI:** 10.1093/gerona/glag090

**Published:** 2026-04-07

**Authors:** Ivan Baltasar-Fernandez, Pedro L Valenzuela, Irene Rodríguez-Gómez, Fabio Quiñonez-Bareiro, Luis M Alegre, Konstantinos Prokopidis, Leocadio Rodriguez-Mañas, Ignacio Ara, Oscar Rosas-Carrasco, Lana J Williams, Robinson Ramirez-Vélez, Julie A Pasco, Guy Hajj-Boutros, Raman Agnihotram, Marc Sim, Wayne Lok Ok Choo, Mahdi Imani, Ana M Ayala-Copete, Julia Chabot, Miguel G Borda, Nailton J Neto, Claire Godard-Sebillotte, Howard Bergman, Anna Andrianova, Hidenori Arai, Pierrette Gaudreau, Liang Kung Chen, Hussein Samhat, Tiago d S Alexandre, Ricardo Guerra, Andréa Faust, Alexandra Papaioannou, Harmehr Sekhon, Emma Connolly, Gustavo Duque, Francisco J García-García, Transforming Health and Resilience for Healthy Lives (THRIVE) Project Group

**Affiliations:** GENUD Toledo Research Group, Faculty of Sport Sciences, University of Castilla-La Mancha, Toledo, Spain; Faculty of Health Sciences, University of Castilla-La Mancha, Talavera de la Reina, Spain; Grupo Mixto de Fragilidad y Envejecimiento Exitoso UCLM-SESCAM, Universidad de Castilla-La Mancha-Servicio de Salud de Castilla-La Mancha, Instituto de Investigación Sanitaria de Castilla-La Mancha (IDISCAM), Toledo, Spain; Centro de Investigación Biomédica en Red Fragilidad y Envejecimiento Saludable (CIBERFES), Instituto de Salud Carlos III, Madrid, Spain; GENUD Toledo Research Group, Faculty of Sport Sciences, University of Castilla-La Mancha, Toledo, Spain; Centro de Investigación Biomédica en Red Fragilidad y Envejecimiento Saludable (CIBERFES), Instituto de Salud Carlos III, Madrid, Spain; Department of Systems Biology, University of Alcalá, Madrid, Spain; GENUD Toledo Research Group, Faculty of Sport Sciences, University of Castilla-La Mancha, Toledo, Spain; Grupo Mixto de Fragilidad y Envejecimiento Exitoso UCLM-SESCAM, Universidad de Castilla-La Mancha-Servicio de Salud de Castilla-La Mancha, Instituto de Investigación Sanitaria de Castilla-La Mancha (IDISCAM), Toledo, Spain; Centro de Investigación Biomédica en Red Fragilidad y Envejecimiento Saludable (CIBERFES), Instituto de Salud Carlos III, Madrid, Spain; Grupo Mixto de Fragilidad y Envejecimiento Exitoso UCLM-SESCAM, Universidad de Castilla-La Mancha-Servicio de Salud de Castilla-La Mancha, Instituto de Investigación Sanitaria de Castilla-La Mancha (IDISCAM), Toledo, Spain; Centro de Investigación Biomédica en Red Fragilidad y Envejecimiento Saludable (CIBERFES), Instituto de Salud Carlos III, Madrid, Spain; Geriatrics Department, Hospital Universitario de Toledo, Toledo, Spain; GENUD Toledo Research Group, Faculty of Sport Sciences, University of Castilla-La Mancha, Toledo, Spain; Grupo Mixto de Fragilidad y Envejecimiento Exitoso UCLM-SESCAM, Universidad de Castilla-La Mancha-Servicio de Salud de Castilla-La Mancha, Instituto de Investigación Sanitaria de Castilla-La Mancha (IDISCAM), Toledo, Spain; Centro de Investigación Biomédica en Red Fragilidad y Envejecimiento Saludable (CIBERFES), Instituto de Salud Carlos III, Madrid, Spain; Department of Musculoskeletal and Ageing Science, Institute of Life Course and Medical Sciences, University of Liverpool, Liverpool, United Kingdom; Centro de Investigación Biomédica en Red Fragilidad y Envejecimiento Saludable (CIBERFES), Instituto de Salud Carlos III, Madrid, Spain; Geriatrics Department, Getafe University Hospital, Getafe, Spain; GENUD Toledo Research Group, Faculty of Sport Sciences, University of Castilla-La Mancha, Toledo, Spain; Grupo Mixto de Fragilidad y Envejecimiento Exitoso UCLM-SESCAM, Universidad de Castilla-La Mancha-Servicio de Salud de Castilla-La Mancha, Instituto de Investigación Sanitaria de Castilla-La Mancha (IDISCAM), Toledo, Spain; Centro de Investigación Biomédica en Red Fragilidad y Envejecimiento Saludable (CIBERFES), Instituto de Salud Carlos III, Madrid, Spain; Geriatric Assessment Center, Health Department, Ibero-American University, Mexico City, Mexico; School of Medicine, Deakin IMPACT-The Institute for Mental and Physical Health and Clinical Translation, Geelong, Victoria, Australia; Navarrabiomed, Ciencias de la Salud, Pamplona, España; School of Medicine, Deakin IMPACT-The Institute for Mental and Physical Health and Clinical Translation, Geelong, Victoria, Australia; Bone, Muscle & Geroscience Group, Research Institute of the McGill University Health Centre, Montreal, Quebec, Canada; Bone, Muscle & Geroscience Group, Research Institute of the McGill University Health Centre, Montreal, Quebec, Canada; Nutrition & Health Innovation Research Institute, Edith Cowan University, Joondalup, Western Australia, Australia; Bone, Muscle & Geroscience Group, Research Institute of the McGill University Health Centre, Montreal, Quebec, Canada; Division of Geriatric Medicine, Department of Medicine, McGill University, Montreal, Quebec, Canada; Bone, Muscle & Geroscience Group, Research Institute of the McGill University Health Centre, Montreal, Quebec, Canada; Division of Geriatric Medicine, Department of Medicine, McGill University, Montreal, Quebec, Canada; RUISS McGill Schouela Centre of Excellence for Sustainable Health of Seniors, Montreal, Quebec, Canada; Bone, Muscle & Geroscience Group, Research Institute of the McGill University Health Centre, Montreal, Quebec, Canada; Geriatric Medicine, Pontificia Universidad Javeriana, Bogotá, Colombia; Division of Geriatric Medicine, Department of Medicine, McGill University, Montreal, Quebec, Canada; Centre for Age-Related Diseases Stavanger, Helse Stavanger HF, Rogaland, Norway; Bone, Muscle & Geroscience Group, Research Institute of the McGill University Health Centre, Montreal, Quebec, Canada; Graduate Program in Health Sciences, Federal University of Rio Grande do Norte, Natal, Brazil; Division of Geriatric Medicine, Department of Medicine, McGill University, Montreal, Quebec, Canada; Division of Geriatric Medicine, Department of Medicine, McGill University, Montreal, Quebec, Canada; RUISS McGill Schouela Centre of Excellence for Sustainable Health of Seniors, Montreal, Quebec, Canada; National Center for Geriatrics and Gerontology, President Office Obu, Aichi, Japan; Department of Medicine, University of Montreal, Montreal, Quebec, Canada; Research Center, Centre Hospitalier de l’Université de Montréal, Montreal, Quebec, Canada; Center for Geriatrics and Gerontology, Taipei Veterans General Hospital, Taipei City, Taiwan; RUISS McGill Schouela Centre of Excellence for Sustainable Health of Seniors, Montreal, Quebec, Canada; Department of Gerontology, Federal University of Sao Carlos, Sao Carlos, Brazil; Graduate Program in Health Sciences, Federal University of Rio Grande do Norte, Natal, Brazil; Bone, Muscle & Geroscience Group, Research Institute of the McGill University Health Centre, Montreal, Quebec, Canada; RUISS McGill Schouela Centre of Excellence for Sustainable Health of Seniors, Montreal, Quebec, Canada; Department of Medicine, McMaster University, Hamilton, Ontario, Canada; Division of Geriatric Medicine, Department of Medicine, McGill University, Montreal, Quebec, Canada; Institute of Neuroscience, Trinity College Dublin, Dublin, Ireland; Bone, Muscle & Geroscience Group, Research Institute of the McGill University Health Centre, Montreal, Quebec, Canada; Division of Geriatric Medicine, Department of Medicine, McGill University, Montreal, Quebec, Canada; RUISS McGill Schouela Centre of Excellence for Sustainable Health of Seniors, Montreal, Quebec, Canada; Grupo Mixto de Fragilidad y Envejecimiento Exitoso UCLM-SESCAM, Universidad de Castilla-La Mancha-Servicio de Salud de Castilla-La Mancha, Instituto de Investigación Sanitaria de Castilla-La Mancha (IDISCAM), Toledo, Spain; Centro de Investigación Biomédica en Red Fragilidad y Envejecimiento Saludable (CIBERFES), Instituto de Salud Carlos III, Madrid, Spain; Geriatrics Department, Hospital Universitario de Toledo, Toledo, Spain; (Medical Sciences Section)

**Keywords:** Bone, Muscle, Older adults, Adverse outcomes, Healthy aging

## Abstract

**Background:**

Osteosarcopenia—the coexistence of osteopenia/osteoporosis and sarcopenia—is associated with numerous adverse health outcomes, but its association with intrinsic capacity (IC) remains unknown. We aimed to explore the associations of osteosarcopenia with IC and all-cause mortality in older adults.

**Methods:**

We conducted a cross-sectional analysis of IC and a prospective analysis of all-cause mortality in 1142 participants from the Toledo Study for Healthy Aging (75.1 ± 6.0 years, 53.1% women). Participants were classified as having no musculoskeletal disorders, osteopenia/osteoporosis, sarcopenia, or osteosarcopenia (DXA- and EWGSOP2-based criteria). IC was operationalized as a composite score integrating locomotion, cognition, psychological, vitality, and sensory domains.

**Results:**

Older adults with osteosarcopenia exhibited lower total IC (76.6 ± 1.1 points) than those without musculoskeletal disorders (83.7 ± 0.5 points; *p *< .001) or with osteopenia/osteoporosis (81.3 ± 0.3 points; *p *< .001), with significant differences also found between the latter groups (*p *< .001). Specifically, osteosarcopenia was associated with poorer results in the locomotion, cognition, vitality, and sensory domains (all *p *< .01), but not in the psychological domain. Over a median 6.2-year follow-up, osteosarcopenia—but not osteopenia/osteoporosis—was associated with a higher risk of mortality compared to those without musculoskeletal disorders [HR (95% CI)=1.96 (1.02-3.77); *p *= .045]. In joint analyses with IC status (high vs low, segregated by the median), this association was specifically observed among participants with osteosarcopenia and low IC [HR = 2.55 (1.31-4.96); *p *= .004].

**Conclusions:**

Osteosarcopenia is associated with lower total IC and impairments across most IC domains. This finding is of clinical relevance, as the combination of this condition and low IC is associated with an increased mortality risk.

## Introduction

Sarcopenia and osteoporosis—defined as an excessive loss of muscle mass/strength and bone mineral density (BMD), respectively—are two prevalent musculoskeletal conditions in older adults, affecting 10%-27%^1^ and ∼22%^2^ of this population, respectively. These conditions are associated with numerous adverse health outcomes, including higher rates of hospitalization, disability, fractures, morbidity, and mortality.^3–5^ In recent years, it has been proposed that there is a crosstalk between bone and muscle, and that with aging, both tissues undergo parallel changes at macroscopic and molecular levels.^6^ These alterations can lead to the coexistence of sarcopenia and osteoporosis in some patients, a geriatric syndrome termed osteosarcopenia that affects 16%-21% of older adults.^7^^,^^8^

Controversy exists on whether osteosarcopenia is associated with a higher risk of adverse health outcomes compared to sarcopenia or osteoporosis alone, although some reports suggest that individuals with osteosarcopenia have a higher mortality risk.^9^ It is worth noting that, although healthy aging has been traditionally defined based on the presence or absence of comorbidities and other adverse health events, in recent years, the World Health Organization (WHO) has proposed a shift from this disease-centered approach to a function-centered one, defining healthy aging as the process of maintaining functional ability over time.^10^

To address this need, the WHO has introduced intrinsic capacity (IC) as a marker of older adults’ overall health, defining it as the composite of an individual’s physical and mental capacities, typically assessed across 5 domains: locomotion, cognition, psychological function, vitality, and sensory function.^11^ IC has indeed proven to be a valid predictor of adverse outcomes, such as disability and mortality.^12^^,^^13^ However, although a recent cross-sectional study showed that osteosarcopenia was associated with impairments in IC,^14^ this topic—and particularly whether this association differs from that of individuals with sarcopenia or osteopenia/osteoporosis alone—remains largely unexplored. The present study aimed to explore the association of osteosarcopenia with IC and all-cause mortality in older adults, as well as to determine whether a low IC could increase the mortality risk associated with osteosarcopenia.

## Methods

### Study design and participants

This study included data from the second wave of the Toledo Study for Healthy Aging (TSHA),^15^ collected between 2011 and 2013, with follow-up through 2019. Participants were originally recruited using a two-stage random sampling stratified by sex, age, and town size.

For the present analyses, we included individuals who had complete data on both IC and body composition parameters at baseline, as well as on mortality status during the follow-up. All participants provided written informed consent prior to participation. The study protocol was approved by the Clinical Research Ethics Committee of the Toledo Hospital Complex (Spain) (July 15, 2010; reference number 93) and was conducted in accordance with the Declaration of Helsinki.

### Anthropometric and body composition parameters

Body mass was measured to the nearest 0.1 kg using a calibrated scale (Seca 711, Hamburg, Germany), and height was measured to the nearest 1 mm using a portable stadiometer (Medizintechnikseit 1890; KaWe, Asperg, Germany). Participants were assessed barefoot while wearing light clothing. Body mass index (BMI) was calculated as body mass divided by height squared (kg·m^−2^).

Body composition was assessed using dual-energy x-ray absorptiometry (DXA) (Hologic Discovery QDR Series, Bedford, MA), and all scans were analyzed using the manufacturer’s software (Physician’s Viewer, APEX System Software Version 3.1.2, Bedford, USA). The device was calibrated daily according to the manufacturer’s recommendations. Participants were positioned supine on the scanning table wearing light, metal-free clothing. A regional analysis was performed to determine appendicular lean mass, defined as the sum of the fat-free soft tissue mass of both arms and legs. The appendicular skeletal muscle index (ASMI) was then calculated by dividing appendicular lean mass by height squared (kg/m^2^). In addition, bone mineral content (g) and BMD (g/cm^2^) were assessed for the whole body and specific regions, including the lumbar spine (L1-L4) and femoral neck (coefficients of variation are 1% for BMC and 1.4% for BMD).^16^ These assessments were performed in a predefined sub-cohort of participants who attended a dedicated laboratory facility for DXA evaluation.

### Musculoskeletal health

Musculoskeletal health was characterized based on bone and muscle status. Participants were classified into 4 mutually exclusive categories: osteopenia/osteoporosis, sarcopenia, osteosarcopenia, or without musculoskeletal disorders.

The presence of osteopenia or osteoporosis (i.e., osteopenia/osteoporosis) was defined according to the WHO criteria.^17^ Specifically, a T-score <−1.0 derived from BMD measurements at either the lumbar spine (L1-L4) or the femoral neck was considered indicative of osteopenia/osteoporosis.

Sarcopenia was defined as the coexistence of low muscle strength and low muscle mass following the criteria by the European Working Group on Sarcopenia in Older People 2 (EWGSOP2).^18^ Low muscle strength was defined as handgrip strength <27 kg for men and <16 kg for women, measured using a Jamar hydraulic dynamometer (5030J1, Jamar, Anaheim, CA). Low muscle mass was defined as ASMI <7.0 kg/m^2^ in men and <5.5 kg/m^2^ in women, derived from DXA measurements.

Osteosarcopenia was defined as the coexistence of both osteopenia/osteoporosis and sarcopenia.^7^ Participants who did not meet the criteria for either osteoporosis-penia or sarcopenia were classified as having no musculoskeletal disorders.

### Intrinsic capacity

In line with the conceptual framework proposed by the WHO,^13^ IC was operationalized as a multidimensional construct comprising 5 domains: locomotion, cognition, psychology, vitality, and sensory.

Following the procedures performed in previous studies,^19^^,^^20^ locomotion was evaluated through the Short Physical Performance Battery (SPPB; score range 0-12, higher is better), which includes walking speed, 5-repetition sit-to-stand, and balance tests.^21^ Cognition was assessed using the Mini-Mental State Examination (MMSE; score range 0-30, higher scores indicating better performance).^22^ Psychology was assessed using the Geriatric Depression Scale (GDS; score range 0-15, higher scores indicating worse depressive symptoms).^23^ Vitality was evaluated using the Mini Nutritional Assessment (MNA; score range 0-30, higher scores indicating better nutritional status),^24^ instead of handgrip strength as proposed in the original framework.^19^ This adaptation was made to avoid collinearity with the sarcopenia and osteosarcopenia definitions, since handgrip strength was already included in their criteria. Importantly, the MNA is one of the variables suggested by the WHO Working Group on Vitality Capacity to assess the vitality domain.^25^ Sensory function was assessed through self-reported vision and hearing performance.

Following previous methodological approaches,^19^^,^^20^ each domain score was rescaled from 0 to 100 points, where 100 represented the best and 0 the worst performance in the original scale. For instance, for the SPPB, a score of 12 corresponded to 100 points, whereas for the GDS, a score of 0 corresponded to 100 points. For the sensory domain, vision and hearing were scored separately from 0 to 50 points (50 = no impairment, 25 = mild impairment, 0 = moderate/severe impairment), and the domain score was calculated as the sum of both components (range 0-100), thus aligning with the rescaling approach used for the other domains. The overall IC score was computed as the mean of the 5 rescaled domain scores (range 0-100 points), with higher values indicating better IC. Only participants with data on all domains were included in the analyses.

### All-cause mortality

All-cause mortality data were retrieved from the Spanish National Death Index (Ministry of Health, Consumer Affairs, and Social Welfare). Follow-up time was calculated from the date of the baseline assessments to the date of death or censoring (December 2019).

### Covariates

Age (years), sex (men/women), educational level, number of medications, and comorbidities were evaluated and included as covariates in the analyses. Educational level was classified into 4 categories: illiterate, primary education, secondary education, and university education. Medication use was assessed by recording the total number of regularly prescribed drugs, which was analyzed as a continuous variable and used as an indicator of polypharmacy. Comorbidities were evaluated using the Charlson Comorbidity Index, which assesses the presence and severity of 19 chronic conditions, providing a weighted summary score that reflects overall comorbidity burden.^26^

### Statistical analysis

Descriptive data are shown as mean ± standard deviation (SD) for continuous variables and frequencies (n) and percentages (%) for categorical variables. Kolmogorov-Smirnov and Levene’s tests were used to evaluate the normality of the distribution and the homoscedasticity of the sample, respectively. One-way ANOVA and *χ*^2^ tests were used to evaluate baseline differences between musculoskeletal health groups in continuous and categorical variables, respectively. Linear mixed models adjusted for age, sex, educational level, number of medications, and comorbidities were used to compare total and domain-specific IC across musculoskeletal health groups, with Bonferroni’s post hoc tests applied to assess between-group differences. Kaplan-Meier survival curves were generated to depict cumulative survival probabilities across musculoskeletal health groups. Subsequently, Cox proportional hazards regression models were used to estimate hazard ratios (HR, along with 95% confidence intervals [95% CI]) for all-cause mortality. We also checked the proportional hazard assumption using log-log survival plots. The same analyses were conducted to analyze the joint association of musculoskeletal health groups and IC status (high [combining the two highest quartiles] vs low [combining the two lowest quartiles]) with mortality risk. All models were adjusted for age, sex, educational level, number of medications, and comorbidities. To assess potential concerns related to multicollinearity, we calculated variance inflation factors (VIFs) for the fully adjusted model (all values <1.2, indicating an absence of relevant multicollinearity). All statistical analyses were performed using SPSS v26 (SPSS Inc., Chicago, IL), and the significance level was set at *α* = .05.

## Results

From the whole cohort (*n* = 2551), after excluding participants with missing body composition (*n* = 712), IC or mortality status data (*n* = 697), a total of 1142 individuals (mean age = 75.1 ± 6.0 years; 536 men and 606 women) were included in the final analytic sample. Baseline characteristics of the study sample are presented in Table 1, while comparisons between included participants and those excluded due to missing DXA data are provided in Table S1. At baseline, participants with osteosarcopenia (*n* = 48, 4.2%) were older and exhibited lower body mass and BMI, poorer physical function (SPPB), lower cognitive function (MMSE), and reduced nutritional status (MNA) compared with those without musculoskeletal disorders (*n* = 265, 23.2%) and those with osteopenia/osteoporosis (*n* = 824, 72.2%) (all comparisons *p *< .05). They also showed a higher prevalence of severe visual and hearing impairment and a greater mortality rate (all comparisons *p *< .05). In contrast, GDS scores and Charlson Comorbidity Index values were similar across groups (all comparisons *p *> .05). Given the low number of participants with sarcopenia alone (*n* = 5, 0.4%), these subjects were removed from subsequent analyses.

**Table 1:** Baseline characteristics of the study sample.

	Total sample (*N* = 1142)	Without MS disorders (*n* = 265)	Osteopenia/osteoporosis (*n* = 824)	Sarcopenia (*n* = 5)	Osteosarcopenia (*n* = 48)
Sex, male, *n* (%)	536 (46.9)	175 (66.0)	328 (39.8)	5 (100.0)	28 (58.3)
Age (years)	75.1 ± 6.0	73.2 ± 4.9	75.4 ± 6.1^a^	79.1 ± 6.3	79.8 ± 7.3^a^^,^^b^
Body mass (kg)	73.8 ± 12.7	81.2 ± 11.1	72.3 ± 12.2^a^	68.7 ± 7.8	59.8 ± 9.7^a^^,^^b^
Height (m)	1.56 ± 0.09	1.61 ± 0.09	1.55 ± 0.09^a^	1.63 ± 0.03	1.54 ± 0.08^a^
BMI (kg/m^2^)	30.3 ± 4.7	31.6 ± 4.4	30.3 ± 4.6^a^	26.0 ± 2.8^a^	25.0 ± 3.1^a^^,^^b^
Charlson index (points)	0.53 ± 0.84	0.55 ± 0.84	0.52 ± 0.83	1.40 ± 0.89	0.60 ± 1.03
SPPB (points)	8.8 ± 2.2	9.3 ± 1.9	8.8 ± 2.2^a^	7.6 ± 2.9	7.4 ± 2.6^a^^,^^b^
MMSE (points)	24.5 ± 4.0	25.2 ± 3.4	24.4 ± 4.1^a^	21.2 ± 4.1	21.9 ± 4.7^a^^,^^b^
GDS (points)	3.3 ± 2.0	3.2 ± 2.0	3.4 ± 2.0	3.8 ± 1.9	2.9 ± 1.9
MNA (points)	24.5 ± 1.7	24.8 ± 1.6	24.5 ± 1.7^a^	24.1 ± 2.1	23.6 ± 1.9^a^^,^^b^
**Visual impairment**					
No impairment, *n* (%)	1059 (92.7)	250 (94.3)	765 (92.8)	4 (80.0)	40 (83.3)^a^^,^^b^
Mild, *n* (%)	70 (6.1)	14 (5.3)	50 (6.1)	1 (20.0)	5 (10.4)
Moderate-severe, *n* (%)	13 (1.2)	1 (0.4)	9 (1.1)	0 (0.0)	3 (6.3)^a^^,^^b^
**Hearing impairment**					
No impairment, *n* (%)	1022 (89.5)	249 (94.0)	730 (88.6)	3 (60.0)	40 (83.3)^a^
Mild, *n* (%)	105 (9.2)	15 (5.7)	85 (10.3)^a^	2 (40.0)	3 (6.3)
Moderate-severe, *n* (%)	15 (1.3)	1 (0.4)	9 (1.1)	0 (0.0)	5 (10.4)^a^

Data are shown as mean ± SD unless otherwise stated.

Abbreviations: BMI, body mass index; GDS, Geriatric Depression Scale; MMSE, Mini-Mental State Examination; MNA, Mini Nutritional Assessment; MS, musculoskeletal; SPPB, Short Physical Performance Battery.

a Significantly different compared to older adults without MS disorders (*p *< .05).

b Significantly different compared to older adults with osteopenia/osteoporosis (*p *< .05).

### Intrinsic capacity across musculoskeletal health status

**Figure 1:**
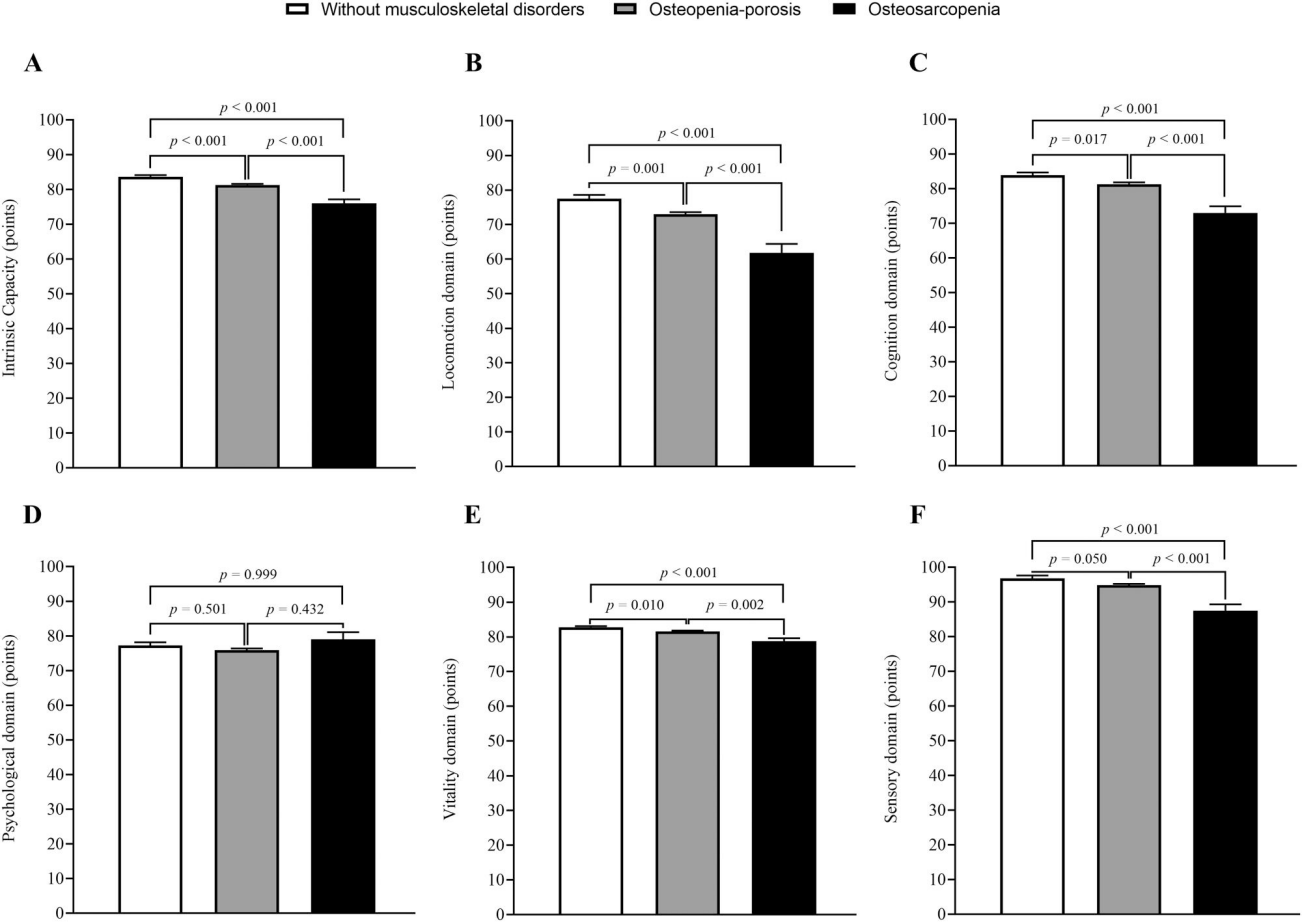
Intrinsic capacity scores across musculoskeletal health groups. Estimated means and standard errors of total intrinsic capacity (IC) and its domain-specific scores are shown, derived from linear mixed models adjusted for age, sex, educational level, number of medications, and comorbidities. Panels show (A) total IC score, (B) locomotion domain, (C) cognition domain, (D) psychological domain, (E) vitality domain, and (F) sensory domain.

Participants with osteosarcopenia exhibited significantly lower total IC scores compared to those without musculoskeletal disorders (76.0 ± 1.1 vs 83.7 ± 0.5 points; *p *< .001) and those with osteopenia/osteoporosis (76.0 ± 1.1 vs 81.3 ± 0.3 points; *p *< .001), with significant differences also observed between the latter groups (*p *< .001) (Figure 1).

In domain-specific analyses, individuals with osteosarcopenia showed significantly lower scores for the locomotion, cognition, vitality, and sensory domains compared to both individuals without musculoskeletal disorders and those with osteopenia/osteoporosis, with significant differences also observed for these domains between individuals with osteopenia/osteoporosis and those without musculoskeletal disorders (Figure 1). In turn, no significant differences were observed in the psychological domain between older adults with osteosarcopenia and those without musculoskeletal disorders or osteopenia/osteoporosis alone, nor between those with osteopenia/osteoporosis and those without musculoskeletal disorders (Figure 1).

### Association between musculoskeletal health and all-cause mortality

**Figure 2:**
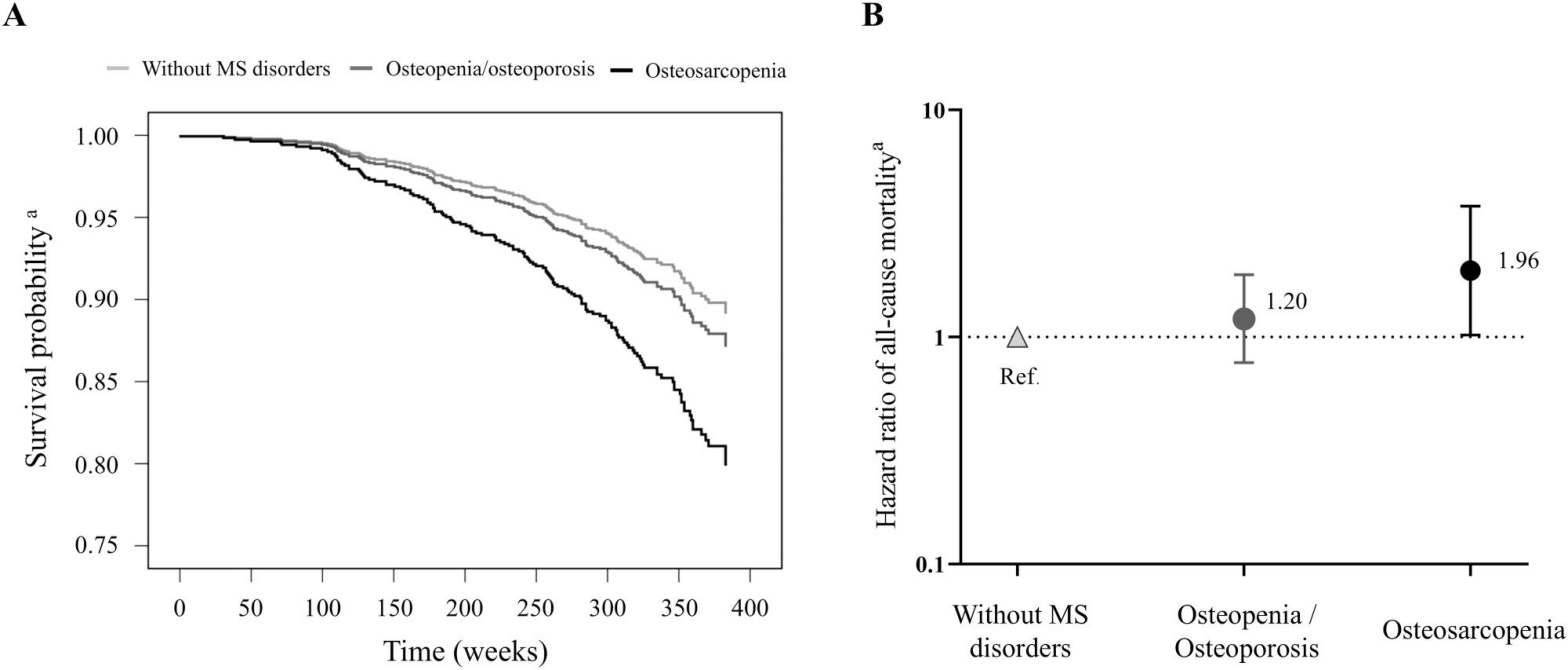
Association between musculoskeletal health and all-cause mortality. Adjusted Kaplan–Meier survival curves (A) and (B) hazard ratios (HRs) derived from Cox proportional regression models showing the association between musculoskeletal health status and all-cause mortality. MS, musculoskeletal; Ref., reference group. ^a^Models were adjusted for age, sex, educational level, number of medications, and comorbidities.

**Figure 3:**
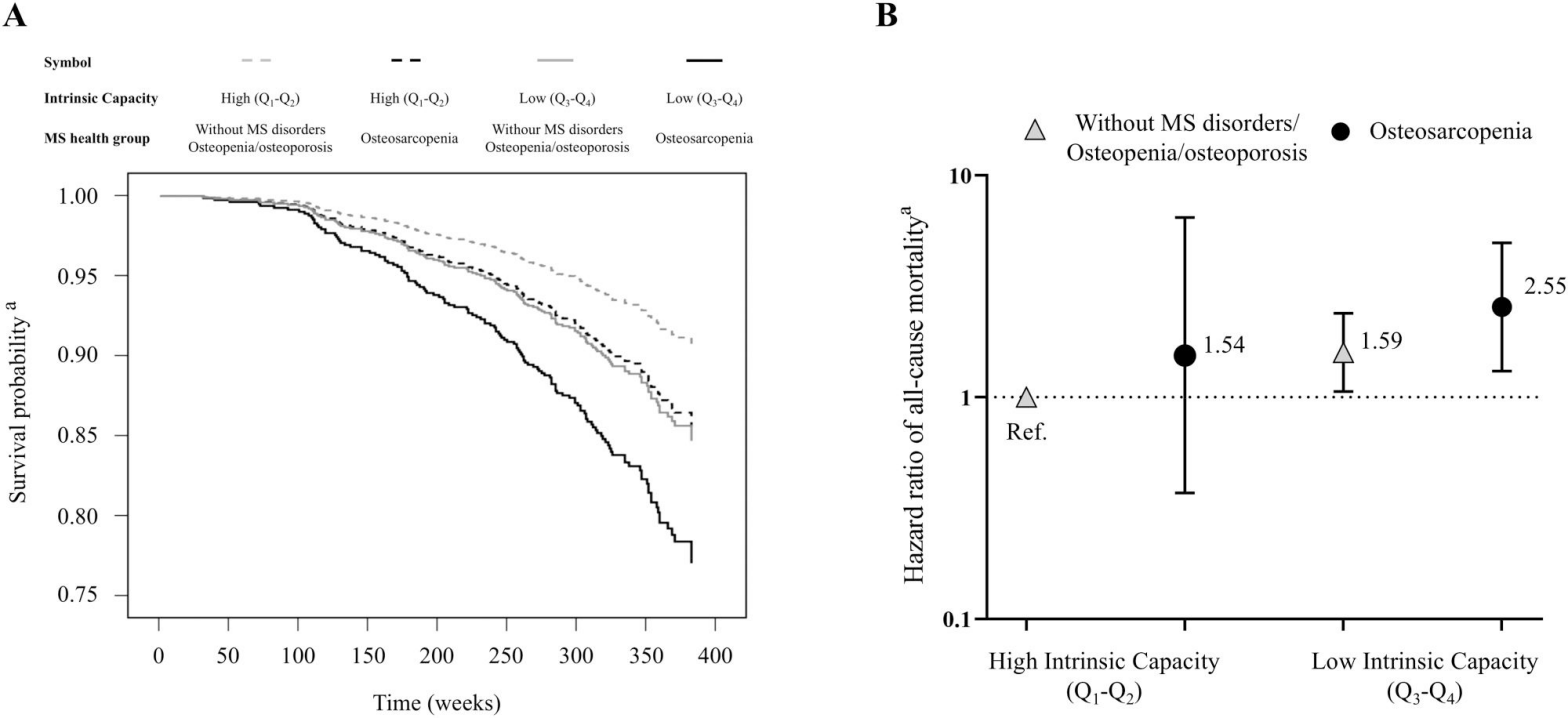
Joint association of musculoskeletal health and intrinsic capacity with all-cause mortality. Adjusted Kaplan–Meier survival curves (A) and (B) hazard ratios (HRs) derived from Cox proportional regression models showing the combined association of musculoskeletal health and intrinsic capacity groups (high IC [combining the two highest quartiles] vs low IC [combining the two lowest quartiles]) with all-cause mortality risk. ^a^Models were adjusted for age, sex, educational level, number of medications, and comorbidities. IC, intrinsic capacity; MS, musculoskeletal; Ref., reference group.

The incidence of mortality was assessed during a median follow-up of 6.2 years. Adjusted Kaplan-Meier survival curves (Figure 2A) and Cox proportional hazards models (Figure 2B) showed that, compared to participants without musculoskeletal disorders, those in the osteopenia/osteoporosis group did not exhibit a statistically significant increase in mortality risk [HR (95% CI) = 1.20 (0.77, 1.88); *p *= .430]. In contrast, participants with osteosarcopenia had a significantly higher risk of all-cause mortality than those without musculoskeletal disorders [HR (95% CI) = 1.96 (1.02-3.77); *p *= .045]. Interestingly, joint analyses of musculoskeletal health status and IC (i.e., low vs high IC) showed that only those participants with osteosarcopenia and low IC had an increased mortality risk compared to those without musculoskeletal disorders or osteopenia/osteoporosis [HR (95% CI) = 2.55 (1.31, 4.96); *p *= .006], whereas this association was not significant in those with osteosarcopenia but high IC [HR (95% CI) = 1.54 (0.37, 6.45); *p *= .559] (Figure 3). A similar finding was observed for individuals without osteosarcopenia (i.e., without musculoskeletal disorders or with osteopenia/osteoporosis), with those with a low IC showing an increased risk of all-cause mortality compared to their counterparts with high IC [HR (95% CI) = 1.59 (1.06, 2.39); *p *= .024].

## Discussion

The main finding of the present study was that individuals with osteosarcopenia showed a reduced IC compared not only to participants without musculoskeletal disorders but also compared to those with osteopenia/osteoporosis alone. This finding was confirmed for most IC domains, including locomotion, cognition, vitality, and sensory, albeit not the psychological domain. This result is of clinical relevance, as we observed that low IC was associated with increased mortality risk in general and particularly among individuals with osteosarcopenia, with those individuals with osteosarcopenia and high IC showing no increased mortality risk compared to those without this condition.

The association between musculoskeletal disorders and IC remains largely unknown, but growing evidence suggests that conditions such as sarcopenia and osteopenia/osteoporosis can independently affect IC.^27^ For instance, a cross-sectional study in hospitalized older adults observed that lower IC scores (particularly in the vitality domain) were associated with sarcopenia, although some specific sarcopenia components were also associated with impairments in individual IC domains (e.g., lower scores in cognition, locomotion, and vitality were associated with weaker handgrip strength).^28^ Another cross-sectional study by the same research group observed that hospitalized older adults with osteoporosis were more likely to present impairments in IC, particularly in the locomotor domain.^29^ Scarcer evidence exists, however, on whether individuals with osteosarcopenia present greater impairments in IC compared to individuals with sarcopenia or osteoporosis alone. To the best of our knowledge, only one study has explored the association between osteosarcopenia and IC. In line with our findings, in a cross-sectional study involving 461 Chinese older adults, Qiao et al. found that individuals with osteosarcopenia had lower total IC scores compared to not only healthy participants, but also to those with osteoporosis or sarcopenia.^14^ This finding is of relevance, as IC has proven to be a prognostic factor of functional disability and mortality.^12^ Indeed, in the present study, we observed that individuals with osteosarcopenia—particularly those with low IC—had a higher mortality risk compared to those with osteopenia/osteoporosis alone, which is in line with previous reports.^9^ Given the higher prevalence of osteoporosis within the osteosarcopenia group compared with the osteopenia/osteoporosis group (50% vs 33.7%), we conducted additional analyses adjusting for osteoporosis status to determine whether differences in bone disease severity could account for our findings. Notably, the results of the joint analyses remained materially unchanged after this additional adjustment, suggesting that the prognostic relevance of intrinsic capacity among individuals with osteosarcopenia may not be fully explained by differences in underlying bone disease severity. Moreover, a recent study reported that osteosarcopenia is associated with a greater risk of functional decline compared to individuals with sarcopenia or osteopenia/osteoporosis alone.^30^ Monitoring and promoting IC among individuals with osteosarcopenia appears, therefore, of paramount importance, although the association might be bidirectional. In this sense, the coexistence of impaired bone and muscle health may directly compromise locomotion, nutritional status, and cognitive reserve through shared biological pathways such as chronic low-grade inflammation, hormonal dysregulation, mitochondrial dysfunction, and reduced mechanotransduction.^6^^,^^31^ On the other hand, declines in IC—particularly in locomotion and vitality—may promote physical inactivity, malnutrition, and anabolic resistance, thereby accelerating musculoskeletal deterioration.^32–35^ This reciprocal interplay may contribute to the progressive vulnerability observed in affected individuals.

When analyzing specific IC domains, in the present study, we observed that participants with osteosarcopenia had lower scores for the locomotion, vitality, cognition, and sensory domains compared to individuals without musculoskeletal conditions and those with osteopenia/osteoporosis. This result is overall in line with the findings by Qiao et al.,^14^ who confirmed that osteosarcopenia was associated with larger impairments in the locomotion, cognition, and vitality domains compared to osteoporosis or sarcopenia, although, as opposed to the present study, no differences were found for the sensory domain. Mechanistically, there is evidence to support these impairments in individuals with osteosarcopenia, as well as in those with sarcopenia or osteopenia/osteoporosis alone. The more clearly affected domain is locomotion, as both reduced muscle strength/mass and reduced BMD limit mobility and increase the risk of falls and fractures.^31^ Similarly, malnutrition, a marker of the vitality domain, is also associated with both sarcopenia^33^ and poor bone health.^34^ However, there is also evidence to support that both sarcopenia and osteoporosis independently contribute to the risk of cognitive impairment.^36^^,^^37^ For instance, the odds of cognitive impairment are nearly threefold higher in sarcopenic adults,^36^ which might be explained by diverse biological mechanisms such as reductions in neurotrophic muscle-derived factors (e.g., brain-derived neurotrophic factor, irisin),^38^ and a low BMD has also been associated with a greater risk of dementia.^37^

An interesting result of the present study was that individuals with osteosarcopenia had similar scores for the psychological domain compared to the other groups. In contrast, Qiao et al. observed that individuals with osteosarcopenia had poorer scores for this domain compared to their healthy peers.^14^ This is an unexpected finding, particularly given that research overall suggests that both sarcopenia and osteoporosis are independently associated with depression.^39^^,^^40^ Indeed, different studies have reported that osteosarcopenia is cross-sectionally and longitudinally associated with the presence and development of depression, even more than in participants with osteoporosis alone.^41^^,^^42^ One possible explanation is the relatively low burden of depressive symptoms observed in our cohort (mean GDS score ∼3 points), which may have limited variability and reduced our ability to detect between-group differences. Alternatively, unmeasured psychosocial factors—such as higher levels of social support or care, which have been previously linked to osteosarcopenia^43^—may partly help explain this unexpected pattern found in our study. Further research is therefore warranted to elucidate the association between osteosarcopenia and mental health outcomes.

Importantly, emerging evidence suggests that declines in IC may be amenable to nonpharmacological interventions such as exercise training.^44^ Multicomponent exercise programs, combining strength, balance, mobility, and aerobic training, have consistently shown benefits across locomotor, cognitive, and vitality domains in older adults in different settings.^45^^,^^46^ However, given the multidimensional nature of IC, intervention strategies should not rely solely on exercise and may also include nutritional interventions, cognitive stimulation, psychological support, and management of sensory impairments. In addition, considering that osteosarcopenia was associated with markedly lower IC in our cohort, strategies targeting both musculoskeletal health and IC may offer additive value. From a clinical perspective, the combined assessment of musculoskeletal health and IC may also improve case finding and risk stratification in older adults. Incorporating IC evaluation within routine clinical practice or Comprehensive Geriatric Assessment could help identify individuals at the highest risk and guide personalized, multidimensional prevention strategies. Future studies should determine whether improving IC can mitigate the adverse outcomes associated with osteosarcopenia, including mortality.

This study has some limitations that should be acknowledged. First, its observational design precludes establishing causality between osteosarcopenia and IC, particularly given the cross-sectional nature of this analysis. Therefore, future longitudinal studies are warranted to determine the directionality of these associations, specifically, whether low IC predisposes individuals to the development of osteosarcopenia or whether osteosarcopenia itself accelerates the decline in IC. Second, as the sample was derived from a Spanish cohort of community-dwelling older adults, the generalizability of the findings may be limited to other countries or populations. Thus, replication in other cohorts and populations is needed to confirm the robustness and external validity of the present results. Third, the relatively small number of participants with sarcopenia restricted the ability to analyze this group separately. Future studies including a larger number of purely sarcopenic individuals are required to elucidate the isolated contribution of poor muscle health and its interaction with bone health to the osteosarcopenia phenotype and healthy aging. Fourth, the limited number of participants with osteosarcopenia reduced statistical power for sex-stratified analyses, and potential sex-specific associations warrant further investigation in larger cohorts. Fifth, sensory function was assessed using self-reported vision and hearing performance rather than objective clinical measures, which may have led to underestimation of sensory deficits. Finally, as DXA assessments were conducted in a dedicated laboratory facility, participation may have required a minimum level of mobility and functional capacity. Consequently, individuals with more severe functional impairment may have been underrepresented in the analytical sample, and the findings may be less generalizable to more frail older populations.

## Conclusions

Osteosarcopenia was associated with lower total IC and a higher mortality risk compared not only to participants without musculoskeletal disorders but also compared to those with osteopenia/osteoporosis alone. This finding was confirmed for most IC domains, including the locomotion, vitality, cognition, and sensory domains. The combined presence of osteosarcopenia and low IC identified individuals at the greatest risk of death, underscoring a synergistic interplay between musculoskeletal deterioration and functional decline. These findings highlight the need for integrative strategies targeting both musculoskeletal health and IC to extend healthy longevity in older adults.

## Supplementary Material

glag090_Supplementary_Data

## Data Availability

The data that support the findings of this study are available from the corresponding author upon reasonable request.

## References

[1] Petermann-Rocha F , Celis-Morales C, Balntzi V, et al Global prevalence of sarcopenia and severe sarcopenia : a systematic review and meta-analysis. J Cachexia Sarcopenia Muscle. 2022;13:86-99. 10.1002/jcsm.12783 34816624 PMC8818604

[2] Salari N , Darvishi N, Bartina Y, et al Global prevalence of osteoporosis among the world older adults : a comprehensive systematic review and meta‑analysis. J Orthop Surg Res. 2021;16:669-613. 10.1186/s13018-021-02821-8 34774085 PMC8590304

[3] Beaudart C , Alcazar J, Aprahamian I, et al; Global Leadership Initiative in Sarcopenia (GLIS) Group. Health outcomes of sarcopenia : a consensus report by the outcome working group of the Global Leadership Initiative in Sarcopenia (GLIS). Aging Clin Exp Res. 2025;37:100. 10.1007/s40520-025-02995-9 40120052 PMC11929733

[4] Pan XB , Ma QY, Gao T, et al Osteoporosis risk and its association with all-cause and cause-specific mortality among the elderly : a 16-year nationwide cohort study. BMC Geriatr. 2025;25:199. 10.1186/s12877-025-05843-7 40140739 PMC11948726

[5] Rodriguez-Gomez I , Gray S, Ho F, et al Osteoporosis and its association with cardiovascular disease, respiratory disease, and cancer: findings from the UK Biobank prospective cohort study. Mayo Clin Proc. 2022;97:110-121. 10.1016/j.mayocp.2021.07.019 34996542

[6] Kirk B , Lombardi G, Duque G. Bone and muscle crosstalk in ageing and disease. Nat Rev Endocrinol. 2025;21:375-390. 10.1038/s41574-025-01088-x 40011751

[7] Hirschfeld HP , Kinsella R, Duque G. Osteosarcopenia: where bone, muscle, and fat collide. Osteoporos Int. 2017;28:2781-2790. 10.1007/s00198-017-4151-8 28733716

[8] Chen S , Xu X, Gong H, et al Global epidemiological features and impact of osteosarcopenia : a comprehensive meta-analysis and systematic review. J Cachexia Sarcopenia Muscle. 2024;15:8-20. 10.1002/jcsm.13392 38086772 PMC10834350

[9] Blomqvist M , Nuotio MS, Sääksjärvi K, Pentti J, Koskinen S, Stenholm S. Osteosarcopenia as a risk factor for fractures and mortality—19-year follow-up of a population-based sample. Aging Clin Exp Res. 2025;37:319-319. 10.1007/s40520-025-03229-8 41196446 PMC12592282

[10] Beard JR , Officer A, De Carvalho IA, et al The world report on ageing and health: a policy framework for healthy ageing. Lancet. 2016;387:2145-2154. 10.1016/S0140-6736(15)00516-4 26520231 PMC4848186

[11] Gonzalez-Bautista E , Llibre-Guerra JJ, Sosa ALA, et al Exploring the natural history of intrinsic capacity impairments: longitudinal patterns in the 10/66 study. Age Ageing. 2023;52:afad137. 10.1093/ageing/afad137 37517058 PMC10387229

[12] Sánchez-Sánchez JL , Lu WH, Gallardo-Gómez D, et al Association of intrinsic capacity with functional decline and mortality in older adults: a systematic review and meta-analysis of longitudinal studies. Lancet Heal Longev. 2024;5:e480-e492. 10.1016/S2666-7568(24)00092-8 38945130

[13] World Health Organization. *Integrated Care for Older People: Guidelines on Community-Level Interventions to Manage Declines in Intrinsic Capacity*; 2017. 10.1007/978-3-319-96529-1_19 29608259

[14] Qiao X , Zhang L, Man F, Wang W, Guo L, Pan Q. New indicators related to the osteosarcopenia in the elderly : assessment of intrinsic capacity. BMC Geriatr. 2025;25:737. 10.1186/s12877-025-06424-4 41023866 PMC12482731

[15] Garcia-Garcia FJ , Gutierrez Avila G, Alfaro-Acha A, et al; Toledo Study Group. The prevalence of frailty syndrome in an older population from Spain. The Toledo study for healthy aging. J Nutr Health Aging.. 2011;15:852-856. 10.1007/s12603-011-0075-8 22159772 PMC12878005

[16] Sutter T , Duboeuf F, Lespessailles E, Roux J P, Chapurlat R, Cortet B. DXA body composition corrective factors between Hologic discovery models to conduct multicenter studies. Bone. 2021;142:115683. 10.1016/j.bone.2020.115683 33045389

[17] Kanis J , Melton L, Christiansen C, Johnston C, Khaltaev N. The diagnosis of osteoporosis. J Bone Miner Res. 1994;9:1137-1141. 10.1002/jbmr.5650090802 7976495

[18] Cruz-Jentoft AJ , Bahat G, Bauer J, et al; Writing Group for the European Working Group on Sarcopenia in Older People 2 (EWGSOP2), and the Extended Group for EWGSOP2. Sarcopenia: revised European Consensus on Definition and Diagnosis. Age Ageing. 2019;48:16-31. 10.1093/ageing/afy169 30312372 PMC6322506

[19] Lu WH , Rolland Y, Guyonnet S, Barreto PDS, Vellas B. Reference centiles for intrinsic capacity throughout adulthood and their association with clinical outcomes: a cross-sectional analysis from the INSPIRE-T cohort. Nat Aging. 2023;3:1521-1528. 10.1038/s43587-023-00522-x 37946044

[20] Stolz E , Mayerl H, Freidl W, Roller-Wirnsberger R, Gill TM. Intrinsic capacity predicts negative health outcomes in older adults editor’s choice. J Gerontol A Biol Sci Med Sci. 2022;77:101-105. 10.1093/gerona/glab279 34569602 PMC8751795

[21] Guralnik JM , Simonsick EM, Ferrucci L, et al A short physical performance battery assessing lower extremity function: association with self-reported disability and prediction of mortality and nursing home admission. J Gerontol. 1994;49:M85-M94. 10.1093/geronj/49.2.m85 8126356

[22] Folstein MF , Folstein SE, McHugh PR. “Mini-mental state”. A practical method for grading the cognitive state of patients for the clinician. J Psychiatr Res. 1975;12:189-198. 10.1016/0022-3956(75)90026-6 1202204

[23] Martínez de la Iglesia J , Onís Vilches MC, Dueñas Herrero R, Albert Colomer C, Aguado Taberné C, Luque R. Versión española del cuestionario de Yesavage abreviado (GDS) para el despistaje de depresión en mayores de 65 años: adaptación y validación. Medifam. 2002;12:26-40. ibc-16673

[24] Vellas B , Guigoz Y, Garry PJ, et al The mini nutritional assessment (MNA) and its use in grading the nutritional state of elderly patients. Nutrition. 1999;15:116-122. 10.1016/s0899-9007(98)00171-3 9990575

[25] Bautmans I , Knoop V, Amuthavalli Thiyagarajan J, et al WHO Working Group on Vitality Capacity. WHO working definition of vitality capacity for healthy longevity monitoring. Lancet Heal Longev. 2022;3:e789-e796. 10.1016/S2666-7568(22)00200-8 PMC9640935 36356628

[26] Charlson M , Szatrowski TP, Peterson J, Gold J. Validation of a combined comorbidity index. J Clin Epidemiol. 1994;47:1245-1251. 10.1016/0895-4356(94)90129-5 7722560

[27] Barbosa Sales W , Vitor de Souza Silva P, Barbosa Vital B, Camara M. Sarcopenia and intrinsic capacity in older adults : a systematic review. Arch Gerontol Geriatr. 2025;135:105875. 10.1016/j.archger.2025.105875 40318296

[28] Zhu L , Zong X, Shi X, Ouyang X. Association between intrinsic capacity and sarcopenia in hospitalized older patients. J Nutr Health Aging. 2023;27:542-549. 10.1007/s12603-023-1946-5 37498101 PMC12929992

[29] Zhu L , Shen X, Shi X, Ouyang X. Factors associated with intrinsic capacity impairment in hospitalized older adults : a latent class analysis. BMC Geriatr. 2024;24:494. 10.1186/s12877-024-05093-z 38840051 PMC11151595

[30] Rosas-Carrasco O , Manrique-Espinoza B, López-Alvarenga JC, Mena-Montes B, Omaña-Guzmán I. Osteosarcopenia predicts greater risk of functional disability than sarcopenia : a longitudinal analysis of FraDySMex cohort study. J Nutr Heal Aging. 2024;28:100368. 10.1016/j.jnha.2024.100368 PMC12879309 39307074

[31] Ferrucci L , Baroni M, Ranchelli A, et al Interaction between bone and muscle in older persons with mobility limitations. Curr Pharm Des. 2014;20:3178-3197. 10.2174/13816128113196660690 24050165 PMC4586132

[32] Gaussens L , González-Bautista E, Bonnefoy M, et al Associations between Vitality/Nutrition and the Other Domains of Intrinsic Capacity Based on Data from the INSPIRE ICOPE-Care Program. Nutrients 2023;15:1567. 10.3390/nu15071567 37049408 PMC10096560

[33] Calcaterra L , Abellan van Kan G, Steinmeyer Z, Angioni D, Proietti M, Sourdet S. Sarcopenia and poor nutritional status in older adults. Clin Nutr. 2024;43:701-707. 10.1016/j.clnu.2024.01.028 38320461

[34] Siddique N , Donoghue MO, Casey MC, Walsh JB. Malnutrition in the elderly and its effects on bone health. A review. Clin Nutr ESPEN. 2017;21:31-39. 10.1016/j.clnesp.2017.06.001 30014867

[35] Sánchez-Sánchez JL , Ortolá R, Banegas JR, et al Articles association between physical activity and sedentary behaviour and changes in intrinsic capacity in Spanish older adults (Seniors-ENRICA-2): a prospective population-based study. Lancet Heal Longev. 2025;6:100681. 10.1016/j.lanhl.2024.100681 40414228

[36] Chang KV , Hsu TH, Wu WT, Huang KC, Han DS. Association between sarcopenia and cognitive impairment: a systematic review and meta-analysis. J Am Med Dir Assoc. 2016;17:1164.e7-1164-e15. 10.1016/j.jamda.2016.09.013 27816484

[37] Kawaguchi K , Maeda M, Murata F, Nakashima Y, Fukuda H. Association of low bone mineral density and dementia in older women : insights from the Longevity Improvement and Fair Evidence Study. Age Ageing. 2025;54:1-9. 10.1093/ageing/afaf058 40100148

[38] Valenzuela PL , Castillo-García A, Morales JS, et al Exercise benefits on Alzheimer’s disease: state-of-the-science. Ageing Res Rev. 2020;62:101108. 10.1016/j.arr.2020.101108 32561386

[39] Kashfi SS , Abdollahi G, Hassanzadeh J, Mokarami H, Jeihooni AK. The relationship between osteoporosis and depression. Sci Rep. 2022;12:11177. 10.1038/s41598-022-15248-w 35778459 PMC9249757

[40] Li Z , Tong X, Ma Y, Bao T, Yue J. Prevalence of depression in patients with sarcopenia and correlation between the two diseases : systematic review and meta-analysis. J Cachexia Sarcopenia Muscle. 2022;13:128-144. 10.1002/jcsm.12908 34997702 PMC8818614

[41] Veronese N , Ragusa FS, Sabico S, et al Osteosarcopenia as a risk factor for depression: Longitudinal findings from the SHARE study. Bone Rep. 2025;25:101848. 10.1016/j.bonr.2025.101848 40495908 PMC12148741

[42] Park KS , Lee GY, Seo YM, Seo SH, Yoo JI. Disability, frailty and depression in the community-dwelling older adults with osteosarcopenia. BMC Geriatr. 2021;21:69. 10.1186/s12877-021-02022-2 33468069 PMC7816500

[43] Shiba T , Sawaya Y, Hirose T, et al Features of older community-dwelling adults with osteosarcopenia requiring support or care. J Phys Ther Sci. 2022;34:341-346. 10.1589/jpts.34.341 35527844 PMC9057679

[44] Izquierdo M , de Souto Barreto P, Arai H, et al Global consensus on optimal exercise recommendations for enhancing healthy longevity in older adults (ICFSR). J Nutr Health Aging. 2025;29:100401. 10.1016/j.jnha.2024.100401 39743381 PMC11812118

[45] Sánchez-Sánchez JL , de Souto Barreto P, Antón-Rodrigo I, et al Effects of a 12-week Vivifrail exercise program on intrinsic capacity among frail cognitively impaired community-dwelling older adults: secondary analysis of a multicentre randomised clinical trial. Age Ageing. 2022;51:afac303. 10.1093/ageing/afad050 36580558 PMC9799251

[46] Valenzuela P , Izquierdo M, Martinez-Velilla N, et al Exercise effects on intrinsic capacity in acutely hospitalised older adults: a pooled analysis of two randomised controlled trials. Age Ageing. 2025;54:afaf082. 10.1093/ageing/afaf082 40188489

