# Supplementary material for: The interplay between osteosarcopenia and intrinsic capacity: insights and associations with all-cause mortality in the Toledo Study for Healthy Aging
Source: J Gerontol A Biol Sci Med Sci. 2026 Apr 7;81(6):glag090. doi: 10.1093/gerona/glag090 (PMC13189856; doi:10.1093/gerona/glag090)
Supplement: glag090_Supplementary_Data [file glag090_supplementary_data.docx]

**The Interplay Between Osteosarcopenia and Intrinsic Capacity: Insights and Associations With All-Cause Mortality in the Toledo Study for Healthy Aging**

**eTable 1.** Baseline characteristics of participants included in the analyses and those excluded due to missing DXA data.

|  | | Included participants  (N = 1142) | | | Excluded participants  (n = 712) | | |
| --- | --- | --- | --- | --- | --- | --- | --- |
| Sex, male, n (%) | | 536 (46.9) | | | 289 (40.6)* | | |
| Age (years) | | 75.1 | ± | 6.0 | 78.6 | ± | 7.0* |
| Body mass (kg) | | 73.8 | ± | 12.7 | 72.3 | ± | 13.9* |
| Height (m) | | 1.56 | ± | 0.09 | 1.54 | ± | 0.09* |
| BMI (kg·m^-2^) | | 30.3 | ± | 4.7 | 30.4 | ± | 5.3 |
| Charlson index (points) | | 0.53 | ± | 0.84 | 0.58 | ± | 0.91 |
| SPPB (points) | | 8.8 | ± | 2.2 | 3.8 | ± | 4.4* |
| MMSE (points) | | 24.5 | ± | 4.0 | 23.1 | ± | 5.0* |
| GDS (points) | | 3.3 | ± | 2.0 | 4.1 | ± | 2.4* |
| MNA (points) | | 24.5 | ± | 1.7 | 23.7 | ± | 2.1* |
| Visual impairment | |  |  |  |  |  |  |
|  | No impairment, n (%) | 1059 (92.7) | | | 599 (85.0)* | | |
|  | Mild, n (%) | 70 (6.1) | | | 86 (12.2)* | | |
|  | Moderate-severe, n (%) | 13 (1.2) | | | 20 (2.8)* | | |
| Hearing impairment | |  | | |  | | |
|  | No impairment, n (%) | 1022 (89.5) | | | 594 (84.6)* | | |
|  | Mild, n (%) | 105 (9.2) | | | 60 (8.5) | | |
|  | Moderate-severe, n (%) | 15 (1.3) | | | 48 (6.8)* | | |
| Data are shown as Mean ± SD unless otherwise stated. BMI: Body Mass Index; GDS: Geriatric Depression Scale; MMSE: Mini-Mental State Examination; MNA: Mini Nutritional Assessment; SPPB: Short Physical Performance Battery.  *Note*: Among participants without DXA data, total N was 705 for visual impairment and 702 for hearing impairment.  * Significantly different from included participants (*p*<0.05). | | | | | | | |
